# Rise-and-fall dynamics reveal a molecular and cellular vulnerability axis in prion-like *α*-synuclein propagation

**DOI:** 10.64898/2026.03.27.714785

**Published:** 2026-03-31

**Authors:** Christoffer G. Alexandersen, Julia K. Brynildsen, Alice Prigent, Massimiliano Tamborrino, Anastasia Mantziou, Kevin Kurgat, Michael X. Henderson, Dani S. Bassett

**Affiliations:** 1 Department of Bioengineering, School of Engineering and Applied Science, University of Pennsylvania, PA, USA; 2 Aligning Science Across Parkinson’s (ASAP) Collaborative Research Network, Chevy Chase, MD; 3 Department of Neurodegenerative Science, Van Andel Institute, Grand Rapids, MI, USA; 4 Department of Statistics, University of Warwick, Warwick, UK; 5 Department of Electrical and Systems Engineering, School of Engineering and Applied Science, University of Pennsylvania, Philadelphia, PA, USA; 6 Department of Psychiatry, Perelman School of Medicine, University of Pennsylvania, Philadelphia, PA, USA; 7 Department of Physics and Astronomy, School of Arts and Sciences, University of Pennsylvania, Philadelphia, PA, USA; 8 Department of Neurology, Perelman School of Medicine, University of Pennsylvania, Philadelphia, PA, USA; 9 Santa Fe Institute, Santa Fe, NM, USA

## Abstract

The spread of misfolded proteins through neuronal circuits is a defining feature of neurodegenerative disease, yet the dynamics underlying this process remain poorly understood. Most studies rely on sparsely sampled datasets that capture spatial patterns of pathology but not their temporal evolution. Here, we analyze longitudinal histopathology measurements of *α*-synuclein pathology across hundreds of brain regions in a mouse model of Parkinson’s disease. Network-based dynamical modeling shows that regional pathology does not simply accumulate but instead follows rise-and-fall trajectories across the brain. The inferred parameter landscape reveals a one-dimensional vulnerability axis along which regions with stronger fall dynamics have greater monoaminergic neuronal composition and higher expression of proteostatic and metabolic genes. This vulnerability axis replicates in an independent histopathological dataset, indicating that its dominant transcriptomic structure is preserved. Together, these results suggest that regional vulnerability collapses onto a low-dimensional molecular and cellular axis defined by rise-and-fall dynamics.

## Introduction

Neurodegenerative diseases are characterized by the accumulation of misfolded proteins in the brain, including tau in Alzheimer’s disease and *α*-synuclein in Parkinson’s disease (PD) [1]. These proteins typically have physiological functions but contribute to neuronal dysfunction and degeneration when misfolded. Pathology does not develop uniformly across the brain. Instead, it follows reproducible spatial progression patterns in which some regions are affected earlier and pathology subsequently appears in a subset of anatomically connected regions [2–4]. These stereotyped patterns suggest that disease spreads through the brain rather than emerging independently across regions.

A leading hypothesis proposes that pathological proteins propagate in a *prion-like* manner, transferring between neurons and forming aggregates in recipient cells [5, 6]. Experimental studies support the view that misfolded *α*-synuclein can spread through connected neural circuits [7–9]. However, connectivity alone does not determine where pathology accumulates. Transcriptional variability, cell type composition, and other biological factors also shape the spatial distribution of pathology [8, 10]. A central challenge is therefore to understand how network-mediated propagation interacts with region-specific vulnerability to produce the observed patterns of disease.

Mathematical models have been used to address this problem. Connectome-based approaches have shown that network structure contains substantial information about the spatial distribution of pathology [8, 11–16]. More recent models incorporate local amplification dynamics and fit trajectories directly to data [17–23], and can reproduce non-monotonic pathology trajectories over time, including phases of decline [24]. However, it remains unclear how variation in these dynamics across regions relates to underlying biological factors. This gap in knowledge limits our ability to identify the biological factors that determine regional vulnerability, since these may be reflected in the temporal dynamics of pathology rather than its final magnitude. To identify these factors, a straightforward approach would be to build models informed by data that resolve regional trajectories over time.

Mouse seeding experiments provide such data. In particular, *α*-synuclein preformed fibril (PFF) models enable quantification of pathology across hundreds of anatomically defined regions over multiple post-injection timepoints [8]. This approach makes it possible to measure how pathology evolves in each region, rather than only where it accumulates. Inspection of these trajectories suggests that pathology does not follow simple monotonic accumulation over time.

Here, we analyze whole-brain histopathology measurements of *α*-synuclein in mouse models of PD from 3 days to 9 months after PFF injection into the dorsal striatum. We use a family of network dynamical systems models that describe the spread of pathology across anatomical connections together with region-specific dynamics. Model classes are then compared based on their ability to reproduce the observed spatiotemporal patterns, showing that pathology burden does not simply accumulate but instead follows rise–and-fall dynamics across the brain.

To interpret the biological basis of these dynamics, we integrate whole-brain gene-expression and cell-type data with the inferred model parameters. Rather than analyzing parameters in isolation, we examine their joint correlational structure across genes. This approach reveals a single dominant one-dimensional axis in gene expression that aligns with regional rise–and-fall dynamics, indicating that regional molecular identity is organized along a common gradient. This axis corresponds to a coordinated transcriptional program: regions with stronger fall dynamics show increased expression of genes involved in energy metabolism, synaptic function, and protein homeostasis pathways. It is also reflected in cellular composition: regions with stronger fall dynamics are enriched for monoamine neurons, particularly in dopaminergic populations. To assess generality, we analyze another histopathology dataset with hippocampal PFF injection. Despite the different seeding location, the same one-dimensional structure is recovered, wherein both molecular and cellular associations are preserved. Together, these results suggest that rise–and-fall dynamics are a fundamental feature of *α*-synuclein pathology, and that regional vulnerability is organized along a common molecular and cellular gradient that reflects these dynamics.

## Results

### High-temporal-resolution mapping of *α*-synuclein pathology across the mouse brain

**Figure 1:**
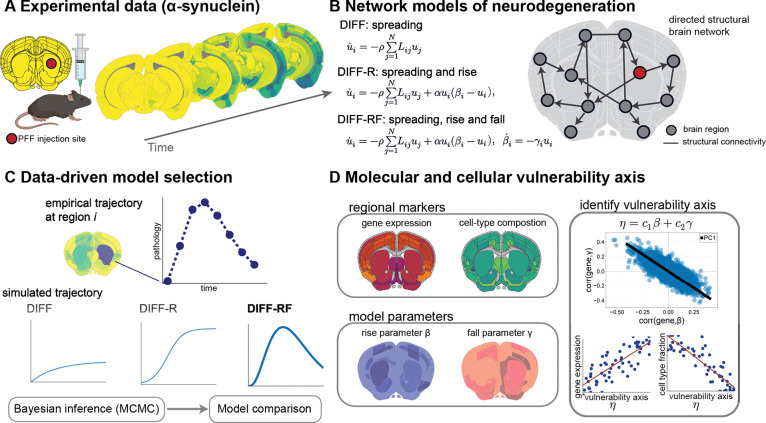
Overview of experimental data, network models, and biological interpretation. (**A**) Experimental α-synuclein seeding paradigm in the mouse brain, illustrating the temporal evolution of pathology following focal injection. (**B**) Network-based dynamical models of neurodegeneration. The DIFF model captures pure network spreading, DIFF-R incorporates local growth or *rise*, and DIFF-RF further includes *fall* dynamics. (**C**) Data-driven model selection. Regional empirical trajectories are compared with simulated dynamics using Bayesian MCMC inference for principled comparison of competing models. (**D**) Molecular and cellular vulnerability axis. Regional gene expression and cell-type composition are related jointly to the rise (β) and fall (γ) parameters. Principal component analysis of gene–parameter associations identifies a dominant axis $\eta =c_{1}\beta +c_{2}\gamma$, and enrichment analysis is performed along this axis to characterize associated pathways and cell types.

We analyzed a high-temporal-resolution histopathology dataset measuring the progression of *α*-synuclein pathology across the mouse brain following focal injection of preformed fibrils (PFFs) into the striatum [15]. Pathology burden was quantified across 412 anatomically defined brain regions, corresponding to nodes in the Allen Mouse Brain Atlas connectome. Measurements were obtained at eight timepoints following injection (0.1, 0.2, 0.3, 0.5, 1, 3, 6, and 9 months), providing a detailed view of disease progression (Fig. 1A).

### Hierarchical dynamical models of *α*-synuclein propagation

We evaluated a family of network-based dynamical models that progressively increase in mechanistic complexity (Fig. 1B). We consider three nested models of pathology spread. The simplest—commonly termed the network *diffusion* model—describes passive spreading of pathology along anatomical connections and is denoted DIFF [11]. The second model augments diffusion with local aggregation dynamics governed by Fisher–Kolmogorov–Petrovsky–Piskunov (FKPP)-type growth, allowing pathology to increase within regions; this formulation has been widely used in prior work, and we denote it DIFF-R, where the ”R” indicates *rise* [17]. The third model further incorporates a process that reduces regional carrying capacity in response to pathology, enabling rise–fall dynamics; we denote this model DIFF-RF, where the ”F” indicates *fall*.

We distinguish between *global* and *regional* model parameters. The DIFF model has one global parameter and no regional parameters. The DIFF-R model has two global parameters and one regional parameter $\beta_{i}\in R$ per brain region, which we refer to as the rise parameter. It determines whether pathology grows locally and its saturation level (the carrying capacity). The DIFF-RF model has two global parameters and two regional parameters $\beta_{i}$ and $\gamma_{i}$. It inherits the rise parameter from DIFF-R and introduces the fall parameter $\gamma_{i}>0$, which controls how rapidly pathology reduces this carrying capacity.

Model parameters were inferred using Bayesian inference with Markov chain Monte Carlo (MCMC), and model classes were compared using likelihood-based information criteria and predictive accuracy metrics, such as the Widely Applicable Information Criterion (WAIC) to select the model that best recapitulates the pathology data (Fig. 1C) [25]. The posterior of the modeling parameters relating to the rise ($\beta_{i}$) and fall ($\gamma_{i}$) were then compared with transcriptomic and cell-type whole-brain data to identify which biological factors associate with the rise and fall dynamics (Fig. 1D). Details of the mathematical models, parameter descriptions, parameter priors, parameter estimation procedures, and transcriptomic and cell-type data are provided in the Materials and Methods.

### Retrograde transport best explains synuclein propagation across the brain

Before comparing dynamical processes, we first examined the direction of protein transport that best explains the data. We considered four candidate mechanisms: retrograde transport, anterograde transport, bidirectional transport (combining retrograde and anterograde), and spatial transport independent of anatomical connectivity (which we dub Euclidean transport).

**Figure 2:**
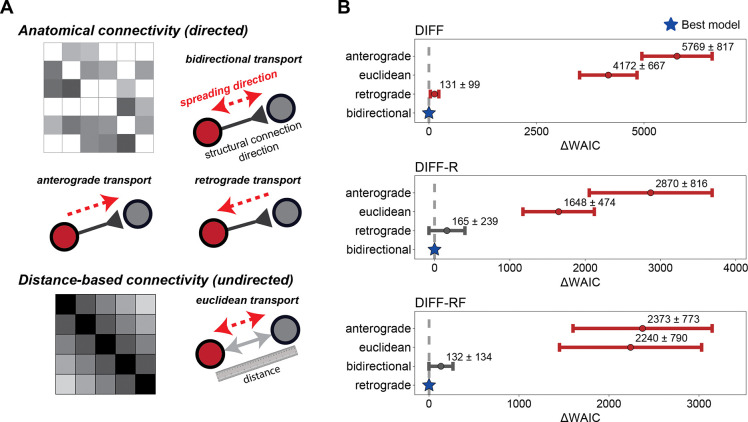
Transport mechanism hypothesis testing. (**A**) Schematic illustration of the weight matrices underlying the transport mechanisms. Anatomical transport operates on a directed structural connectivity matrix, with spreading constrained to existing axonal projections (retrograde, anterograde, or bidirectional). Euclidean transport instead uses a symmetric, distance-based weight matrix independent of anatomical connectivity. (**B**) ΔWAIC (mean ± SD across data points) for four candidate transport mechanisms—retrograde, anterograde, bidirectional, and spatial (Euclidean)—within the DIFF, DIFF-R, and DIFF-RF model classes. Lower values indicate better model performance; stars denote the best-performing mechanism within each class (ΔWAIC = 0,dashed line). Grey lines indicate that the WAIC distribution overlaps with the best performing model.

For each model class, parameters were inferred under each transport mechanism and model performance was compared using WAIC. Across all three model classes, retrograde and bidirectional transport consistently outperform anterograde and Euclidean transport (Fig. 2). Retrograde and bidirectional transport perform similarly across the models, with bidirectional transport narrowly outperforming retrograde transport in the DIFF model. In the DIFF-R and DIFF-RF models, retrograde and bidirectional transport are statistically indistinguishable. These results indicate that α-synuclein propagation is best explained by transport along anatomical connections, with a dominant retrograde component. As such, we continue using a retrograde transport mechanism in all subsequent computational modeling and analyses.

### Prion-like propagation is best captured by rise-and-fall dynamics

**Figure 3:**
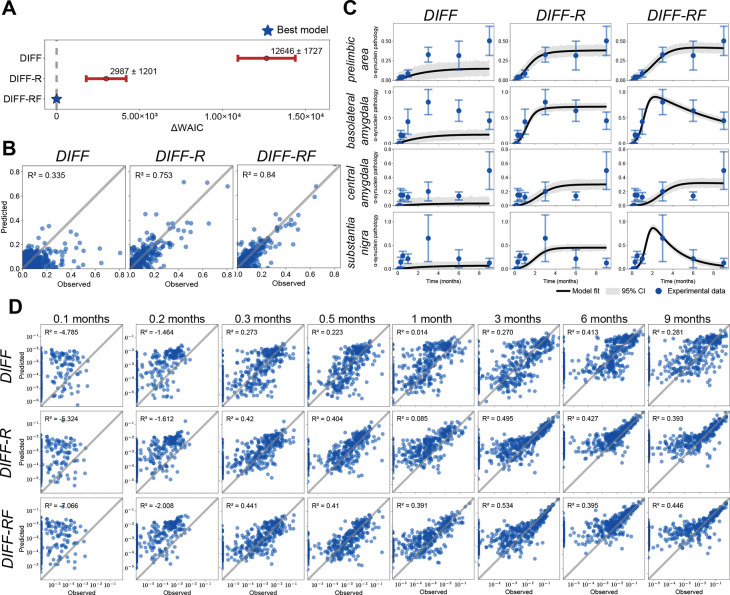
Model comparison and predictive performance across time. (**A**) Model comparison using the WAIC for DIFF, DIFF-R, and DIFF-RF. Points show mean ± standard deviation across data samples, with values reported relative to the best-performing model (ΔWAIC = 0, dashed line). (**B**) Predicted versus observed pathology for each model, pooling all regions and timepoints. Each point corresponds to a region–timepoint pair; the diagonal indicates identity. (**C**) Regional trajectory fits for the four highest-burden regions. Black lines denote model predictions with shaded bands indicating 95% credible intervals; blue points and error bars show empirical mean and standard deviation. (**D**) Log-log predicted versus observed pathology at individual timepoints (all regions pooled). Panels correspond to increasing time after injection (0.1 to 9 months). The diagonal indicates identity.

We next asked which dynamical processes can reproduce the spatiotemporal trajectories observed in the α-synuclein pathology data. To address this question, we compared the three hierarchical model classes introduced above. Model comparison strongly favors rise-and-fall dynamics. As shown in Fig. 3A, DIFF-RF achieves the lowest WAIC, followed by DIFF-R and DIFF. This ordering is consistent across additional metrics (Table 1): DIFF-RF yields the lowest Akaike information criterion and mean squared error, whereas DIFF-R attains the lowest Bayesian information criterion, likely due to its smaller parameter dimension.

We next examined predictive agreement between model-predicted and observed pathology using maximum *a posteriori* parameter estimates and $R^{2}$ computed from regression constrained to pass through the origin. When pooling all regions and timepoints (Fig. 3B), predictive accuracy increases substantially across the model hierarchy, with $R^{2}=0.34$ for DIFF, 0.75 for DIFF-R, and 0.84 for DIFF-RF. These differences are particularly evident in regional trajectory comparisons (Fig. 3C). DIFF produces slow monotonic increases and underestimates peak pathology levels. DIFF-R captures the rapid initial rise and regional saturation but fails to reproduce the subsequent decline observed in several high-burden regions. In contrast, DIFF-RF captures both the rise and the later reduction in pathology where present, while maintaining close agreement in regions that show weaker decline.

The advantage of DIFF-RF is also evident when examining predictions at individual timepoints (Fig. 3D). To capture broad, overall agreement with data across time, we examine the log-log agreement between observations and model predictions. All models show weak agreement at the very earliest time points at 3 and 6 days post injection, but improve rapidly from 9 days onward. DIFF shows weak agreement at early stages and moderate agreement across the time course. DIFF-R and DIFF-RF substantially improve predictive performance, with DIFF-RF consistently reaching higher $R^{2}$ across all time points, excluding at 6 months where DIFF and DIFF-RF perform better. Comparisons in identity (not log-transformed) space give a similar picture, where DIFF-RF consistently outperforms the other models at individual time points (Fig. S1)

Together, these results indicate that pathology does not simply accumulate across the brain but instead follows rise–and-fall trajectories in many regions. Spreading and local growth alone cannot reproduce these dynamics; accurate recovery of the observed trajectories requires an additional region-specific fall process. This observation raises the possibility that the inferred rise and fall parameters reflect underlying biological differences in regional vulnerability.

### Connectome topology and seed location determine propagation dynamics

**Figure 4:**
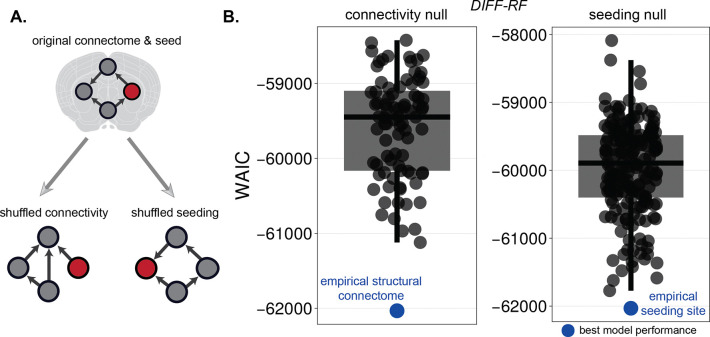
Null-model analysis of connectome topology and seeding location. (**A**) Schematic illustration of the empirical configuration and null-model perturbations. The empirical model uses the anatomical connectome and experimental seeding location. Two null variants are constructed by randomizing either the network connectivity (connectivity null) or the seeding location (seeding null), while keeping all other components unchanged. (**B**) WAIC values for the null models. Grey points show WAIC across randomized realizations; boxes indicate median and interquartile range. The blue point denotes the empirical configuration.

To test whether the model captures propagation in a structurally meaningful way, we evaluated its dependence on the empirical connectome and seeding location. Specifically, we compared the DIFF-RF model using the true structural connectivity and empirical seed region with variants in which either the connectivity matrix was randomly rewired or the seed location was randomized. Model performance was highest when using the empirical connectome and seed region (Fig. 4). Randomizing either the network topology or the seeding location consistently reduced agreement between model predictions and the observed pathology trajectories. These results indicate that accurate reproduction of the data depends critically on both the structure of the connectome and the anatomical origin of pathology.

### Rise-and-fall dynamics improve out-of-sample prediction

**Figure 5:**
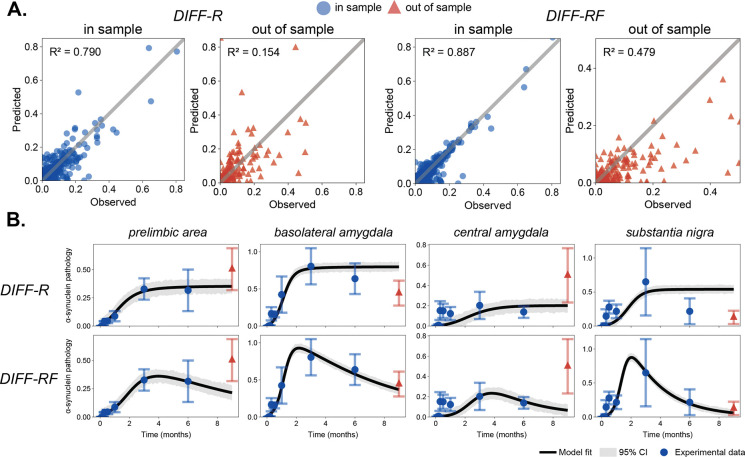
Short-horizon out-of-sample evaluation. (**A**) Predicted versus observed regional pathology for DIFF-R and DIFF-RF. Left panels show agreement across all timepoints, including the held-out observations; right panels show performance on the held-out final timepoint (T−1) only. Red triangles denote held-out observations. DIFF-RF achieves higher $R^{2}$ both overall and on the held-out timepoint. (**B**) Regional trajectories for the four highest-burden regions, with the final timepoint excluded from fitting. Black lines show model predictions; blue circles denote fitted data; red triangles denote held-out observations. DIFF-RF more closely captures the held-out pathology levels.

To assess predictive generalization, we performed a leave-one-timepoint-out analysis in which the final timepoint (T−1) was excluded from model fitting and used for evaluation. Models were fit to the remaining timepoints, and predictions were generated for the held-out observations. Across all regions and timepoints (including the held-out observations), DIFF-RF shows higher predicted–observed agreement than DIFF-R (Fig. 5A, left), with $R^{2}=0.764$ for DIFF-RF compared to $R^{2}=0.599$ for DIFF-R. When evaluated specifically on the held-out timepoint, predictive performance decreases for both models, but DIFF-RF retains substantially higher explanatory power ($R^{2}=0.479$) than DIFF-R ($R^{2}=0.154$) (Fig. 5A, right). Thus, although one-step-ahead prediction is challenging in this dataset, the advantage of incorporating region-specific fall dynamics persists out of sample. Notably, this improvement is also evident in regional trajectory comparisons (Fig. 5B). DIFF-RF more closely matches the held-out pathology values in high-burden regions, whereas DIFF-R tends to over- or under-shoot the final timepoint depending on the region. Together, these results show that incorporating rise-and-fall dynamics improves short-horizon predictive accuracy.

### Inferred dynamical parameters reveal a molecular and cellular vulnerability axis

To interpret the biological processes associated with the inferred dynamical parameters, we examined how regional gene expression relates jointly to the rise (β) and fall (γ) parameters. For each gene, we fit a multiple regression model relating regional expression levels to the standardized, z-transformed parameters z(β) and z(γ). This procedure yields a pair of coefficients describing how strongly each gene’s expression varies with the two rise-and-fall parameters across brain regions. Because fall dynamics can only manifest in regions that permit initial pathological growth, the analyses described below were restricted to regions with positive rise parameters (β>0). Moreover, we omit regions for which the posterior of the regional parameters were not updated from its prior, leading to 77 brain regions in total. We confirmed that the results outlined below are robust with respect to these processing steps (Fig. S2).

**Figure 6:**
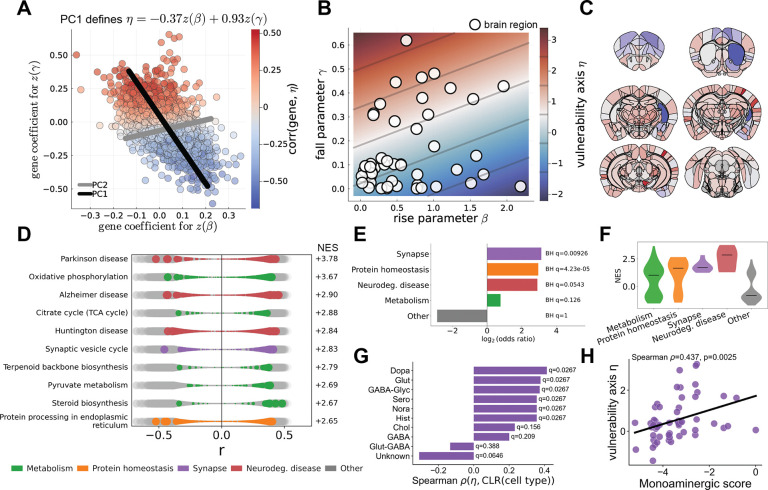
PCA-derived vulnerability axis and biological associations. (**A**) Principal component analysis of gene-level regression coefficients for rise (β) and fall (γ). Each point represents a gene in the space of coefficients for z(β) and z(γ). Black lines denote the first and second principal components (PC1 and PC2). The first principal component defines the axis η. Points are colored by the correlation between gene expression and η. (**B**) Relationship between rise (β) and fall (γ) parameters across brain regions. Points indicate regions, colored by η; background shading shows isocontours of the vulnerability axis. (**C**) Spatial distribution of the vulnerability axis across representative brain sections, showing regional variation in η. (**D**) Gene set enrichment analysis of correlations with the vulnerability axis. Each row shows the distribution of gene-level correlations within a pathway; colored points indicate pathway membership, with normalized enrichment scores (NES) shown on the right. (**E**) Enrichment of functional gene categories along the vulnerability axis, shown as log_2_ odds ratios with Benjamini–Hochberg (BH) adjusted *q*-values. (**F**) Distribution of normalized enrichment scores (NES) across functional categories. (**G**) Association between the vulnerability axis and cell-type signatures, quantified using the Spearman correlation between η and CLR-transformed cell-type expression. (**H**) Relationship between vulnerability and monoaminergic cell-type scores across regions, with Spearman correlation shown.

Each gene can be represented as a point in a two-dimensional coefficient space defined by its correlational associations with z(β) and z(γ) (Fig. 6A). Principal component analysis of this coefficient space revealed a dominant axis capturing the majority of the variance across genes. The first principal component explained 85.5% of the variance and corresponded to the direction η=−0.37z(β)+0.93z(γ), indicating a joint transcriptional gradient associated with lower rise and higher fall parameters (Fig. 6B,C).

To interpret the biological processes associated with this axis, we ranked genes by their correlation (Pearson r) with η and performed pre-ranked gene set enrichment analysis. Genes aligned with increasing η were strongly enriched for pathways related to neurodegenerative disease, synaptic transmission, protein homeostasis, and energy metabolism (Fig. 6D,E,F), including synaptic vesicle cycling, oxidative phosphorylation, and neurotransmitter-related pathways. Genes positively aligned with this axis (η) correspond to regions exhibiting stronger fall and weaker rise dynamics, indicating that synaptic, proteostasis, and metabolic transcriptional programs are upregulated along the direction of increasing γ and decreasing β.

We next examined whether this molecular axis was reflected in regional cellular composition. Using an independent whole-brain cell-type atlas, we related neurotransmitter-class fractions to the regional values of η [26]. Regions with higher η—corresponding to stronger fall dynamics and lower rise—showed higher fractions of monoaminergic neurons (Spearman ρ=0.44,p=0.0025), with the strongest association observed for dopaminergic neurons (Fig. 6G,H).

Together, these results indicate that the inferred parameter landscape is organized along a single molecular and cellular vulnerability axis. Regions enriched in synaptic and proteostasis transcriptional programs and populated by monoaminergic neuronal populations tend to exhibit rise-and-fall dynamics. This pattern suggests that vulnerability is not associated with maximal pathological growth, but with the temporal evolution of pathology, in which burden both accumulates and subsequently declines, defining a characteristic dynamical signature of regional susceptibility.

### Molecular and cellular vulnerability axis replicates across seeding locations

To test whether the molecular and cellular vulnerability axis identified above generalizes across experimental conditions, we analyzed another whole-brain histopathology dataset in which α-synuclein preformed fibrils were injected into the hippocampus (Fig. S3). Pathology was measured across the brain at 4, 6, and 9 months post-injection. We fit the DIFF-RF model to this dataset and obtained strong agreement between predicted and observed regional pathology ($R^{2}=0.88$ for pooled regions and timepoints; Fig. S4).

**Figure 7:**
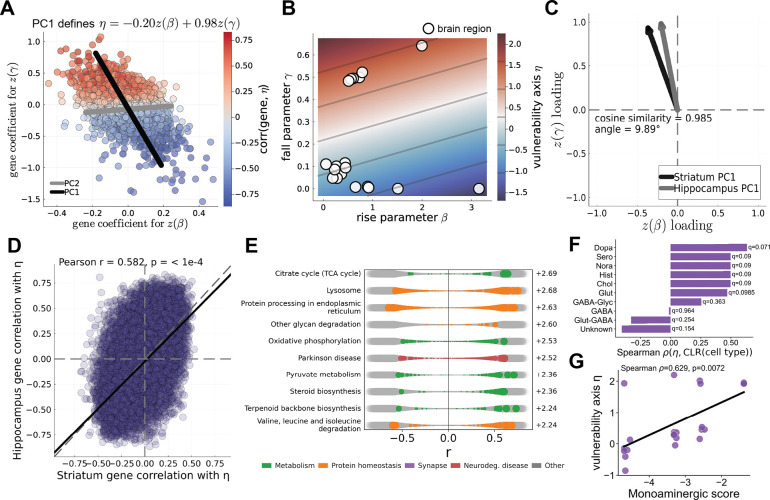
Replication of the PCA-derived vulnerability axis in hippocampal seeding. (**A**) Principal component analysis of gene-level regression coefficients for rise (β) and fall (γ) in the hippocampal dataset. Each point represents a gene; PC1 defines the vulnerability axis η. (**B**) Relationship between rise (β) and fall (γ) parameters across brain regions following hippocampal seeding. Points indicate regions, colored by η, showing the same rise-and-fall structure as in the striatal dataset. (**C**) Comparison of PC1 directions between striatal and hippocampal datasets. (**D**) Concordance of gene-level associations with the vulnerability axis across datasets. Each point shows the correlation of a gene with η in the striatal dataset (x-axis) versus the hippocampal dataset (y-axis). (**E**) Gene set enrichment analysis of correlations with the vulnerability axis in the hippocampal dataset. Each row shows the distribution of gene-level correlations within a pathway; colored points indicate pathway membership, with NES shown on the right. (**F**) Association between the vulnerability axis and cell-type signatures, quantified using Spearman correlation between η and CLR-transformed cell-type expression. (**G**) Relationship between vulnerability and monoaminergic cell-type scores across regions.

We next repeated the joint transcriptomic analysis relating regional gene expression to the inferred rise (β) and fall (γ) parameters. As in the striatal dataset, each gene was represented by its regression coefficients with respect to z(β) and z(γ), and principal component analysis was used to summarize the dominant structure of this coefficient space. The hippocampal dataset again revealed a clear one-dimensional structure. The first principal component explained 87.5% of the variance in gene coefficients and defined the axis η=−0.20z(β)+0.98z(γ), indicating a transcriptional gradient aligned with increasing fall dynamics and decreasing rise (Fig. 7A,B). Thus, despite the different seeding location, the dominant molecular axis again corresponds to the same rise-and-fall dynamical direction identified in the striatal dataset.

To directly compare the two experiments, we next examined the alignment of the transcriptomic coefficient structure across datasets. In the standardized (z(β),z(γ)) coefficient space, the first principal components from the striatal and hippocampal analyses are highly similar (cosine similarity = 0.99, angle = 9.89°; Fig. 7C), while explaining nearly identical fractions of the variance (87.7% and 87.5%, respectively). In the raw (β,γ) coefficient space, the first principal components were nearly collinear (cosine similarity = 0.999, angle = 2.96°; Fig. S5). At the level of individual genes, correlations with the axis η were also preserved across datasets (Pearson $r=0.582,p<10^{-4}$, OLS slope 0.93, 80% sign agreement; Fig. 7D), indicating that the dominant transcriptomic gradient is reproducible across seeding locations.

Pre-ranked gene set enrichment analysis along this axis reproduced the same major biological themes observed previously. Genes aligned with increasing η were enriched for pathways related to energy metabolism, protein homeostasis, and neurodegenerative disease, including oxidative phosphorylation, the citrate cycle, and Parkinson disease pathways (Fig. 7E, Fig. S6). These results recapitulate the enrichment of metabolic and proteostasis transcriptional programs identified in the striatal experiment. Although the hippocampal vulnerability axis was also enriched for synaptic programs, the correlation coefficients were more often related to down-regulation, as opposed to up-regulation in the striatal dataset (Fig. S6). The cell-type analysis yielded a similar replication. Regional values of η were again positively associated with monoaminergic neuronal composition (Spearman ρ=0.63,p=0.0072), and dopaminergic neurons again showed the strongest individual associations among neurotransmitter classes (Fig. 7F,G).

Cross-dataset comparisons show that transcriptional associations with the fall parameter are more robust than those with the rise parameter. Gene coefficients associated with γ showed strong correspondence across datasets (Pearson $r=0.666,p<10^{-4}$, OLS slope 1.18, 79% sign agreement; Fig. S7A), whereas β-associated coefficients were substantially less reproducible (Pearson $r=0.233,p<10^{-4}$, OLS slope 0.33, 57% sign agreement; Fig. S7B). Together, these results show that the vulnerability axis replicates across seeding locations as a joint (β,γ) transcriptional gradient, with its stability primarily driven by transcriptomic structure associated with fall dynamics.

## Discussion

Our results show that α-synuclein pathology follows rise-and-fall trajectories across the brain, and that incorporating this temporal structure is necessary to reproduce regional dynamics. Models that include only spreading and rise capture early accumulation but fail to account for the subsequent decline observed in high-burden regions. This data indicates that regional vulnerability is not determined solely by the capacity to accumulate pathology, but by the temporal evolution of pathology over time. These findings extend connectome-based models of prion-like propagation by showing that temporal trajectories impose additional constraints on model dynamics, consistent with recent wholebrain imaging and modeling studies reporting biphasic pathology trajectories [24]. Here, we show that these dynamics are regionally structured and organized along a low-dimensional axis linked to transcriptional programs and cell-type composition.

A plausible interpretation of the fall parameter is that it reflects neuronal loss following initial accumulation of α-synuclein pathology. Experimental seeding studies show that pathological α-synuclein induces progressive neurodegeneration, including neuritic degeneration and loss of vulnerable neuronal populations [27, 28]. In this context, a reduction in measured pathology is expected if affected neurons or their projections are lost. Network-based analyses further support this interpretation, showing that model-derived vulnerability aligns with gene programs regulating neuronal survival and that perturbation of these pathways reduces both pathology and neuron loss [15]. Together, these results support interpreting fall dynamics primarily as degeneration of vulnerable neuronal populations.

Fall dynamics are unlikely to reflect a single mechanism. Whole-brain modeling integrating gene expression identifies later phases of reduced pathology attributed to local processes beyond spreading [24]. Intracellular degradation pathways, including the autophagy–lysosomal and ubiquitin–proteasome systems, modulate α-synuclein levels in vivo [29, 30], and microglia contribute to extracellular aggregate clearance [31]. These processes can reduce pathology independently of neuronal loss. Fall dynamics should therefore be interpreted as a phenomenological description of decline reflecting a combination of degeneration and clearance processes.

The transcriptional analysis reveals that regional variation in rise and fall dynamics is organized along a one-dimensional axis in gene expression space. This structure arises at the level of gene associations, not the parameters themselves. The rise and fall parameters vary independently across regions, but the genes associated with these parameters align along a common axis. Prior work has linked network-derived vulnerability to regional gene expression, identifying transcriptional correlates of susceptibility [15, 32–34]. In the present results, these associations are organized along a low-dimensional axis that emerges from the joint structure of rise and fall dynamics. The vulnerability axis therefore represents a distinct organizational feature of regional molecular variation associated with propagation dynamics.

The enriched transcriptional programs provide a mechanistic interpretation of this axis. Regions with stronger fall dynamics are associated with genes involved in energy metabolism, protein homeostasis, and synaptic function. Mitochondrial dysfunction increases susceptibility to α-synuclein aggregation and toxicity [35], while disruption of proteostasis alters aggregation dynamics *in vivo* [29]. Synaptic activity modulates α-synuclein release and transmission, and pathological aggregation impairs synaptic function and promotes synapse loss [36, 37]. These findings link fall dynamics to processes governing aggregation, propagation, and degeneration.

The association with monoaminergic neuronal composition provides a complementary cellular interpretation. Regions with stronger fall dynamics are enriched for monoaminergic neurons, particularly dopaminergic populations. Dopaminergic neurons exhibit autonomous pacemaking and sustained calcium influx, leading to mitochondrial oxidant stress and increased vulnerability [38]. Similar vulnerability extends to other monoaminergic systems: noradrenergic neurons develop α-synuclein pathology and dysfunction in targeted models [39], and serotonergic neurons exhibit neuritic degeneration following α-synuclein expression [40]. Manipulating noradrenergic activity alters dopaminergic neuron survival in synucleinopathy models [41]. These results support interpreting the vulnerability axis as reflecting broader monoaminergic susceptibility.

The replication of this axis across striatal and hippocampal seeding datasets indicates that it reflects intrinsic regional properties rather than the site of pathology initiation. Despite differences in seeding location and propagation patterns, the same gene–parameter structure is recovered, and transcriptional associations with fall dynamics are more reproducible than those with rise. This suggests that fall dynamics are more tightly linked to intrinsic biological factors, whereas rise dynamics depend more strongly on seeding location and other experimental factors.

These results also clarify the role of transport directionality. Experimental and modeling studies consistently support a strong retrograde component in α-synuclein propagation, with connectivity-based predictions improving when retrograde projections are emphasized [8, 9, 42]. While anterograde spread can occur, the overall pattern across studies is consistent with retrograde-dominated transport. The present results align with this interpretation.

Several limitations should be noted. The model operates on regionally averaged data and does not resolve cell-type or compartment-specific heterogeneity. It assumes a fixed structural connectome and does not account for connectivity changes induced by pathology. Moreover, the model is deterministic and does not capture the increase in variance of pathology across regions at later timepoints. Finally, it does not incorporate neuronal activity, despite evidence that activity-dependent processes influence α-synuclein propagation and that coupling activity to spreading can alter system dynamics [43–45].

In summary, we identify rise-and-fall dynamics as a fundamental feature of α-synuclein propagation and show that regional vulnerability is encoded in the temporal structure of pathology, with fall dynamics providing a dominant and reproducible signature linked to monoaminergic neuronal composition and specific transcriptional programs.

## Materials and Methods

### Striatal seeding pathology dataset

We analyzed previously published whole-brain histopathology data from mouse models of α-synuclein propagation following unilateral striatal injection of α-synuclein preformed fibrils (PFFs) [15]. Pathology burden was quantified using pS129 α-synuclein immunostaining and registered to the Allen Mouse Brain Atlas CCFv3, yielding region-wise measurements across anatomically defined brain regions. In the present study, we used the regional pathology data aggregated to the brain regions included in the connectome model. Measurements were available at eight post-injection timepoints (0.1, 0.2, 0.3, 0.5, 1, 3, 6, and 9 months). Details of animal procedures, tissue processing, staining, image segmentation, atlas registration, and pathology quantification are provided in the original publication [15].

### Hippocampal seeding pathology dataset

#### Immunohistochemistry

Tissue was obtained from previously described mice [46]. All slides were deparaffinized with two sequential xylene baths (5 min each) and then incubated for 1 minute in a descending series of ethanol baths: 100%, 100%, 95%, 80%, 70%. After a rinse in distilled water, antigen retrieval was performed using citric acid (pH = 6; Vector Laboratories, Cat# H-3300) for 15 min at 95°C. Slides were allowed to cool for 20 min at room temperature and washed in running tap water for 10 min.

To quench endogenous peroxidase activity, slides were incubated in 7.5% hydrogen peroxide for 30 min at room temperature. Slides were washed for 10 min in running tap water, placed for 5 min in 0.1 M Tris buffer (pH = 6), and then blocked for 1 hour at room temperature in 0.1 M Tris with 2% fetal bovine serum (FBS). Slides were incubated overnight at 4°C in primary antibody diluted in 0.1 M Tris/2% FBS in a humidified chamber.

The following primary antibody was used: rabbit polyclonal anti-pS129 α-synuclein (Abcam, Cat# ab51253, RRID:AB_869973). Primary antibodies were rinsed off with 0.1 M Tris for 5 min and then incubated with the appropriate secondary antibody: goat anti-rabbit biotinylated IgG (1:1000; Vector, Cat# BA1000) in 0.1 M Tris/2% FBS for 1 hour at room temperature.

Slides were rinsed using 0.1 M Tris for 5 min, then incubated with avidin–biotin solution (Vector, Cat# PK-6100) for 1 hour. Slides were then rinsed for 5 min with 0.1 M Tris and developed with ImmPACT DAB peroxidase substrate (Vector, Cat# SK-4105). Slides were counterstained for 15 seconds with Harris hematoxylin (Fisher, Cat# 67–650-01).

Slides were washed in running tap water for 5 min, dehydrated in ascending ethanol baths (70%, 80%, 95%, 100%, 100%; 1 min each), and incubated in two sequential xylene baths (5 min each). Slides were mounted with coverslips using Cytoseal mounting media (Fisher, Cat# 23–244-256). Slides were scanned at 20× magnification using an Aperio ScanScope XT. Digitized images were used for quantitative pathology.

### Mouse Brain Segmentation and Registration

#### Segmentation

Scanned slides were imported into QuPath v0.5.0 (RRID: SCR_018257) [47] for analysis. A pixel classifier thresholding fluorescent intensity for the pS129 α-synuclein channel was applied to each section to detect positive pathology signal. Signal intensity was optimized by adjusting min/max display settings, and a consistent threshold was applied across cohorts.

#### Mouse Coronal Section Brain Registration

Images were registered to the Allen Brain Atlas CCFv3 using a modified QUINT workflow (RRID: SCR_023856) [48]. RGB images of each section were exported from QuPath as PNG files and downsampled by a factor of 12 for spatial registration. Segmentation images were generated by exporting color-coded classified pixels or cells on a white background for use in Nutil (RRID: SCR_017183) [49].

Brain images were uploaded to DeepSlice (RRID: SCR_023854) [50], where a deep neural network was used to automatically align sections to atlas regions. Preliminary alignment was performed in QuickNII (RRID: SCR_016854) [51]. Following registration, a JSON file was exported for use in VisuAlign (RRID: SCR_017978).

In VisuAlign, brain sections were refined using nonlinear transformations. Anchor points were placed on the atlas overlay and adjusted to corresponding locations in the tissue sections. Outer contours were aligned first, followed by refinement of internal structures. Final alignments were exported as FLAT and PNG files for use in Nutil.

Nutil was used for quantification and spatial analysis of classified cell types across brain regions. Each segmentation was processed using input masks from QuPath, JSON anchoring files from QuickNII, and transformation files from VisuAlign. Outputs included object area, region area, and area occupied for each classification within regions of the Allen Brain Atlas CCFv3. These outputs were used for downstream modeling and visualization.

### Anatomical Analysis Heatmaps

#### Nutil-to-Usable

Nutil-to-Usable (N2U) is an R-based Shiny application developed at the Van Andel Institute for generating anatomical heatmaps based on the Allen Brain Atlas (RRID: SCR_024753). Outputs from Nutil were uploaded separately for left and right hemispheres.

An annotation file was generated to label metadata for each section (mouse ID, sex, treatment, post-injection interval, etc.). Checkpoint files combining raw Nutil outputs and metadata were used to accelerate downstream processing.

Settings were defined to specify the level of summary and variable of interest. “Load”, defined as the ratio of pathology area to region area, was used as the primary variable when generating heatmaps of total pathology and cell body inclusion area.

### Structural connectivity

Structural connectivity was obtained from the Allen Mouse Brain Connectivity Atlas as described previously [8, 15]. We used the directed projection matrix defined on N=412 Allen CCFv3 brain regions, where each element $W_{ij}$ represents the strength of axonal projections from region j to region i.

To assess whether model performance depends specifically on anatomical connectivity, we also considered a spatial null model based on Euclidean distance between brain regions. In this control model, the connectivity matrix was replaced by a symmetric weight matrix constructed from inter-regional Euclidean distances between region centroids, following the procedure used in previous work [15]. This distance-based matrix preserves the spatial embedding of brain regions while removing anatomical connectivity structure.

### Gene expression data

Regional gene-expression data were obtained from the mouse spatial transcriptomic dataset reported in Ref. [15]. Expression profiles were provided for a subset of brain regions defined in the Allen Mouse Brain Atlas CCFv3. The dataset includes measurements for 132 regions in a single hemisphere.

To align transcriptomic data with hemisphere-specific model parameters, gene-expression values were duplicated across hemispheres, assigning identical expression profiles to the left and right instances of each region. This enabled direct comparison with hemisphere-specific parameter estimates without averaging across hemispheres.

All gene–parameter analyses were performed at the hemisphere level, after matching regions between the transcriptomic dataset and the connectome model.

### Cell-type composition data

Regional cell-type composition was estimated using the Allen Whole Mouse Brain (WMB) cell taxonomy [26]. This dataset defines transcriptomic cell clusters across the mouse brain using single-cell RNA sequencing together with spatial transcriptomic mapping to the Allen Common Coordinate Framework (CCFv3) [26]. Each cluster is annotated by neurotransmitter class and associated with spatial frequency maps across anatomical regions.

Cluster sizes were obtained from the reported number of cells assigned to each transcriptomic cluster. Regional cell counts were then estimated by distributing the cells of each cluster across brain regions according to the reported spatial frequencies. These estimated counts were aggregated across clusters belonging to the same neurotransmitter class to obtain regional counts for each cell type.

When spatial frequencies were reported for anatomical structures that did not exactly match the connectome regions, contributions were propagated through the Allen brain ontology so that descendant regions contributed to their parent structures. This procedure produced estimated cell counts for every connectome region included in the model.

Regional cell-type fractions were computed by normalizing the estimated counts of each neurotransmitter class by the total estimated number of cells in that region. These fractions were used as measures of regional cell-type composition in subsequent analyses.

### Mathematical modeling

#### DIFF: Spreading-only

Let $W\in R^{N\times N}$ denote the directed weighted adjacency matrix of the mouse structural connectome, where $W_{ij}$ represents the strength of the projection from region j to region i. We then define the out-degree graph Laplacian

(1) $$L_{ij}=-W_{ij}+\delta_{ij}\sum_{k=1}^{N}W_{ki},$$

where $\delta_{ij}$ is the Kronecker delta and $\sum_{k}W_{ki}$ corresponds to the total outgoing projection strength from region i. The spreading-only (linear diffusion) model is given by

(2) $${\dot{u}}_{i}=-\rho \sum_{j=1}^{N}L_{ij}u_{j},\;i=1,\ldots ,N,$$

where $u_{i}(t)$ denotes pathology burden in region i and ρ>0 is the transport timescale.

For anterograde transport, the directed connectivity matrix was used as defined above. For retrograde transport, the transpose of the connectivity matrix was used. Bidirectional transport was modeled by adding the spreading processes $\dot{u}=-\rho_{r}L_{r}u-\rho_{a}L_{a}u$, and Euclidean transport was modeled by substituting the anatomical connectivity matrix with a symmetric distance-based weight matrix.

#### DIFF-R: Spreading and rise

However, prion-like spreading is characterized not only by diffusive spreading but also by local aggregation processes. A commonly used model for prion-like aggregation is the heterodimer system,

(3) $$\frac{dp}{dt}=k_{0}-k_{1}p-k_{2}p\tilde{p},$$

(4) $$\frac{d\tilde{p}}{dt}=-k_{3}\tilde{p}+k_{2}p\tilde{p},$$

where $k_{0}>0$ is the protein production rate, $k_{1}>0$ and $k_{3}>0$ are clearance rates, and $k_{2}>0$ is the conversion rate from healthy to misfolded protein [17]. For $p\gg \tilde{p}$, this system can be approximated by Fisher–KPP dynamics for the toxic protein $u=\tilde{p}$,

(5) $$\frac{du}{dt}=\alpha u\left(\beta -u\right),$$

where $\alpha =k_{0}k_{2}^{2}/k_{1}^{2}$ denotes the global aggregation timescale and $\beta =\alpha^{-1}\left(k_{0}k_{2}-k_{1}k_{3}\right)$ is the carrying capacity. We then obtain the spreading-and-rise model

(6) $${\dot{u}}_{i}=-\rho \sum_{j=1}^{N}L_{ij}u_{j}+\alpha u_{i}\left(\beta_{i}-u_{i}\right),\;i=1,\ldots ,N,$$

where $\beta_{i}$ is the region-specific carrying capacity.

#### DIFF-RF: Spreading, rise, and fall

To capture the rise-and-fall pattern observed in the pathology data, we introduce a dynamic carrying capacity that decreases in response to local pathology burden:

(7) $${\dot{u}}_{i}=-\rho \sum_{j=1}^{N}\;L_{ij}u_{j}+\alpha u_{i}\left(\beta_{i}\left(t\right)-u_{i}\right),\;i=1,\ldots ,N,$$

(8) $${\dot{\beta }}_{i}=-\gamma_{i}u_{i},\;i=1,\ldots ,N,$$

where $\gamma_{i}\geq 0$ is the region-specific fall parameter. This additional process allows the local carrying capacity to decrease over time and produces rise-and-fall dynamics in pathology burden. A similar system was proposed to study dynamic clearance mechanisms in prion-like spreading models [52].

In the DIFF-R model, $\beta_{i}$ is a region-specific parameter representing the local carrying capacity. In the DIFF-RF model, $\beta_{i}(t)$ is a state variable. Model inference for DIFF-RF is therefore performed on the initial condition $\beta_{i}(0)$, which we denote by $\beta_{i}$ for notational simplicity.

### MCMC Bayesian estimation of modeling parameters

We inferred the parameters of the mathematical models using Bayesian Markov chain Monte Carlo, which has been used previously to parameterize prion-like propagation models [19, 20, 23, 53, 54]. The prior distributions are summarized in Table 2.

The initial condition is set with all regions set to zero, except for a set of initial seeding regions for which we infer a shared initial value. For the striatal dataset, the initial seeding regions was the ipsilateral striatum region, while for the hippocampal dataset the initial seeding sites were the ipsilateral CA1, CA3, and dentate gyrus.

As the DIFF model conserves total mass, we need a prior which is able to support a large enough initial condition to have enough mass to distribute throughout the network. This is not necessary in DIFF-R and DIFF-RF. As such, the prior for the initial conditions in the DIFF model is considerably wider than in the DIFF-R and -RF models.

Let $y_{ijt}$ denote the observed pathology burden for sample j in region i at timepoint t, and let $u_{i}(t,\theta )$ denote the corresponding model solution. We assumed a normal likelihood with shared observation noise σ,

(9) $$y_{ijt}∼N\left(u_{i}\left(t,\theta \right),\sigma^{2}\right).$$

Posterior distributions were estimated using the No-U-Turn Sampler implemented in Turing.jl, with 1000 warmup iterations, 1000 posterior samples, target acceptance ratio 0.65 with four chains run in parallel. Convergence was assessed using the Gelman–Rubin statistic $\overset{ˆ}{R}$ across chains (Fig. S8). For the models included in the main analyses, most parameters had $\overset{ˆ}{R}<1.05$, with a small number of exceptions. Prior–posterior comparisons for global parameters and pairwise comparisons among regional parameters are shown in Fig. S9. The priors for the global parameters of the DIFF-RF for the hippocampal data are set equal to the posteriors of the striatal dataset, whilst the regional parameters have the same priors as for the striatal dataset.

### Posterior update filtering

For downstream biological analyses, we restricted our attention to regional parameters whose posterior distributions differed significantly from their priors. For each hemisphere-specific parameter, posterior and prior samples were compared using a one-sample Kolmogorov–Smirnov test. Parameters were classified as updated if the Kolmogorov–Smirnov test p-value was less than α=0.001. The main transcriptomic and cell-type analyses were performed on this filtered set of updated parameter estimates.

### Gene–parameter association and enrichment analysis

To interpret the biological processes associated with the regional rise and fall parameters, we analyzed associations between regional gene expression and the inferred parameter maps from the DIFF-RF model. Analyses were restricted to regions with pathology growth ($\beta_{i}>0$) and parameters were classified as updated based on posterior–prior comparison, ensuring that only reliably inferred regional variation contributed to downstream results. For each gene, we fit a multiple linear regression model across regions of the form

(10) $${\text{expression}}_{g}=a_{g}z\left(\beta \right)+b_{g}z\left(\gamma \right)+\varepsilon ,$$

where z(β) and z(γ) denote standardized regional parameter values and ($a_{g},b_{g}$) are gene-specific regression coefficients. This regression was performed across hemisphere-specific observations, yielding one coefficient pair per gene. Each gene is therefore represented as a point in the two-dimensional coefficient space ($a_{g},b_{g}$).

To summarize the dominant structure of this coefficient space, we performed principal component analysis (PCA) across genes. Specifically, PCA was applied to the matrix of gene coefficients [($a_{g},b_{g}$)], treating genes as observations and regression coefficients as variables. The first principal component defined a scalar regional axis

(11) $$\eta =c_{1}z\left(\beta \right)+c_{2}z\left(\gamma \right),$$

where ($c_{1},c_{2}$) are the loadings of the first principal component. For each gene, we then computed the Pearson correlation between its regional expression profile and η. Genes were ranked by this correlation coefficient, which quantifies alignment with the vulnerability axis. Gene set enrichment analysis was performed using pre-ranked GSEA (gseapy) with the KEGG mouse pathway library (KEGG_2019_Mouse). Gene sets containing between 10 and 500 genes were included, and enrichment significance was assessed using 1000 permutations to obtain normalized enrichment scores (NES). Rather than focusing on enrichment significance alone, we examined the distribution of gene-level correlations within each pathway, allowing visualization of how pathway-associated genes align with the vulnerability axis.

For higher-level interpretation, KEGG pathways were grouped into broad functional categories reflecting biological processes commonly implicated in neurodegeneration (metabolism, protein homeostasis, synaptic function, neurodegenerative disease, and other). Assignment was performed using keyword-based matching of pathway names (Table S1). Over-representation of significantly enriched pathways within each category was assessed using Fisher’s exact test, with Benjamini–Hochberg correction applied to control for multiple comparisons.

### Cell-type composition analysis

To relate regional cell-type composition to the inferred model parameters, we analyzed associations between neurotransmitter class fractions and the regional dynamical parameters of the DIFF-RF model. Because cell-type fractions are compositional, fractions were transformed using the centered log-ratio (CLR) transform prior to statistical analysis [55]. Cell-type associations were evaluated primarily with respect to the regional axis η defined by the principal component analysis described above, which summarizes the dominant joint variation of the rise and fall parameters. As for the gene-parameter association analysis, analyses were restricted to regions with pathology growth ($\beta_{i}>0$) and parameters classified as updated based on posterior–prior comparison.

Spearman correlations were computed between CLR-transformed cell-type components and the regional values of η. Hemisphere-specific parameter estimates were retained as separate observations, while cell-type predictors were shared across hemispheres for each region.

Statistical significance was assessed using permutation tests that preserve the mapping between hemispheres belonging to the same region. In each permutation, region identities were shuffled while maintaining the shared predictor values for the two hemispheres of each region. Empirical p-values were obtained from 10,000 permutations. The false positive rate due to multiple testing across cell types was controlled using the Benjamini–Hochberg FDR.

To test whether monoaminergic populations were specifically associated with the vulnerability axis *η*, we defined a monoaminergic score for each region as the mean of the CLR-transformed fractions of dopaminergic, noradrenergic, serotonergic, and histaminergic neurons.

## Supplementary Material

Supplement 1

## Figures and Tables

**Table 1:** Relative model comparison metrics.

Model	ΔWAIC	ΔAIC	ΔBIC	ΔMSE

DIFF	18048	13936	9395	3.22 × 10^−3^
DIFF-R	2961	1326	0	3.20 × 10^−4^
DIFF-RF	0	0	1880	0

All Δ values are computed with respect to the best-performing model for each criterion (minimum value). Lower values indicate better performance.

**Table 2:** Prior distributions for model parameters.

Model	Parameter(s)	Prior

DIFF	ρ	${𝒩}^{+}(50,10)$
DIFF-R	ρ	${𝒩}^{+}(0,0.1)$
	α	${𝒩}^{+}(0,0.1)$
	$\beta_{i}(i=1,\ldots ,K)$	𝒩(0,1)
DIFF-RF	ρ	${𝒩}^{+}(0,0.1)$
	α	${𝒩}^{+}(0,0.1)$
	$\beta_{i}(i=1,\ldots ,K)$	${𝒩}^{+}(0,0.1)$
	$\gamma_{i}(i=1,\ldots ,K)$	${𝒩}^{+}(0,0.1)$

**The index** i=1,…,K **represents brain regions.** 𝒩(μ,σ) **denotes a normal distribution and** ${𝒩}^{+}(\mu ,\sigma )$ **denotes a normal distribution truncated to** (0, ∞).

## Data Availability

Code used for this study is available at https://github.com/gretarsson/synuclein_spread.
