# Supplementary material for: Rise-and-fall dynamics reveal a molecular and cellular vulnerability axis in prion-like α-synuclein propagation
Source: bioRxiv. 2026 Mar 31:2026.03.27.714785. Preprint. [Version 1] doi: 10.64898/2026.03.27.714785 (PMC13060082; doi:10.64898/2026.03.27.714785)
Supplement: Supplement 1 [file media-1.pdf]

Supplementary information for Rise-and-fall dynamics reveal a molecular and cellular vulnerability axis in prion-like α-synuclein propagation

Christoffer G. Alexandersen[1,2], Julia K. Brynildsen[1,2], Alice Prigent[2,3], Massimiliano Tamborrino[4], Anastasia Mantziou[4], Kevin Kurgat[2,3], Michael X. Henderson[2,3], and Dani S. Bassett[1,2,5–9]

[1]Department of Bioengineering, School of Engineering and Applied Science, University of Pennsylvania, PA, USA
[2]Aligning Science Across Parkinson's (ASAP) Collaborative Research Network, Chevy Chase, MD
[3]Department of Neurodegenerative Science, Van Andel Institute, Grand Rapids, MI, USA
[4]Department of Statistics, University of Warwick, Warwick, UK
[5]Department of Electrical and Systems Engineering, School of Engineering and Applied Science, University of Pennsylvania, Philadelphia, PA, USA
[6]Department of Psychiatry, Perelman School of Medicine, University of Pennsylvania, Philadelphia, PA, USA
[7]Department of Physics and Astronomy, School of Arts and Sciences, University of Pennsylvania, Philadelphia, PA, USA
[8]Department of Neurology, Perelman School of Medicine, University of Pennsylvania, Philadelphia, PA, USA
[9]Santa Fe Institute, Santa Fe, NM, USA

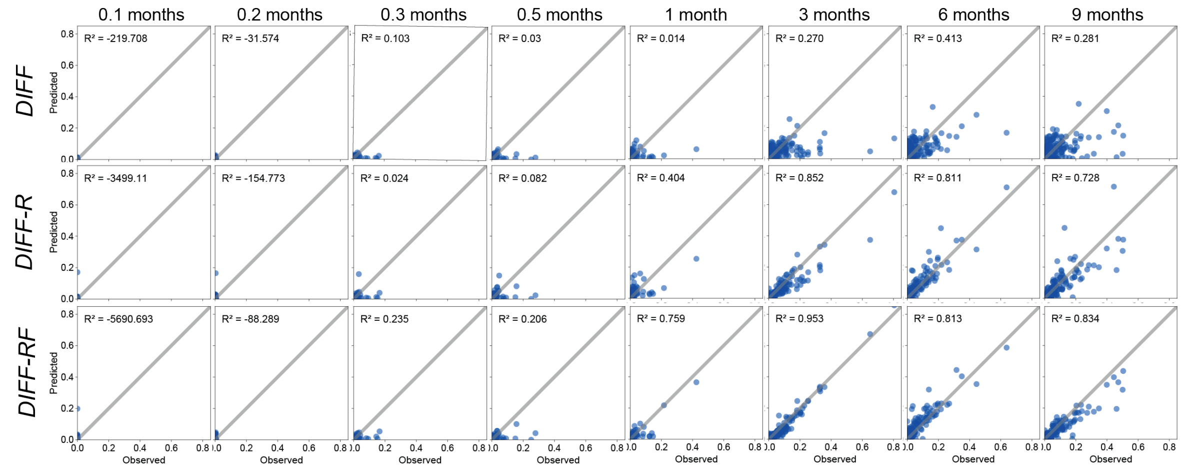

Figure 1: Model predictive performance across time. Predicted versus observed pathology at individual timepoints (all regions pooled). Panels correspond to increasing time after injection (0.1 to 9 months). The diagonal indicates identity.

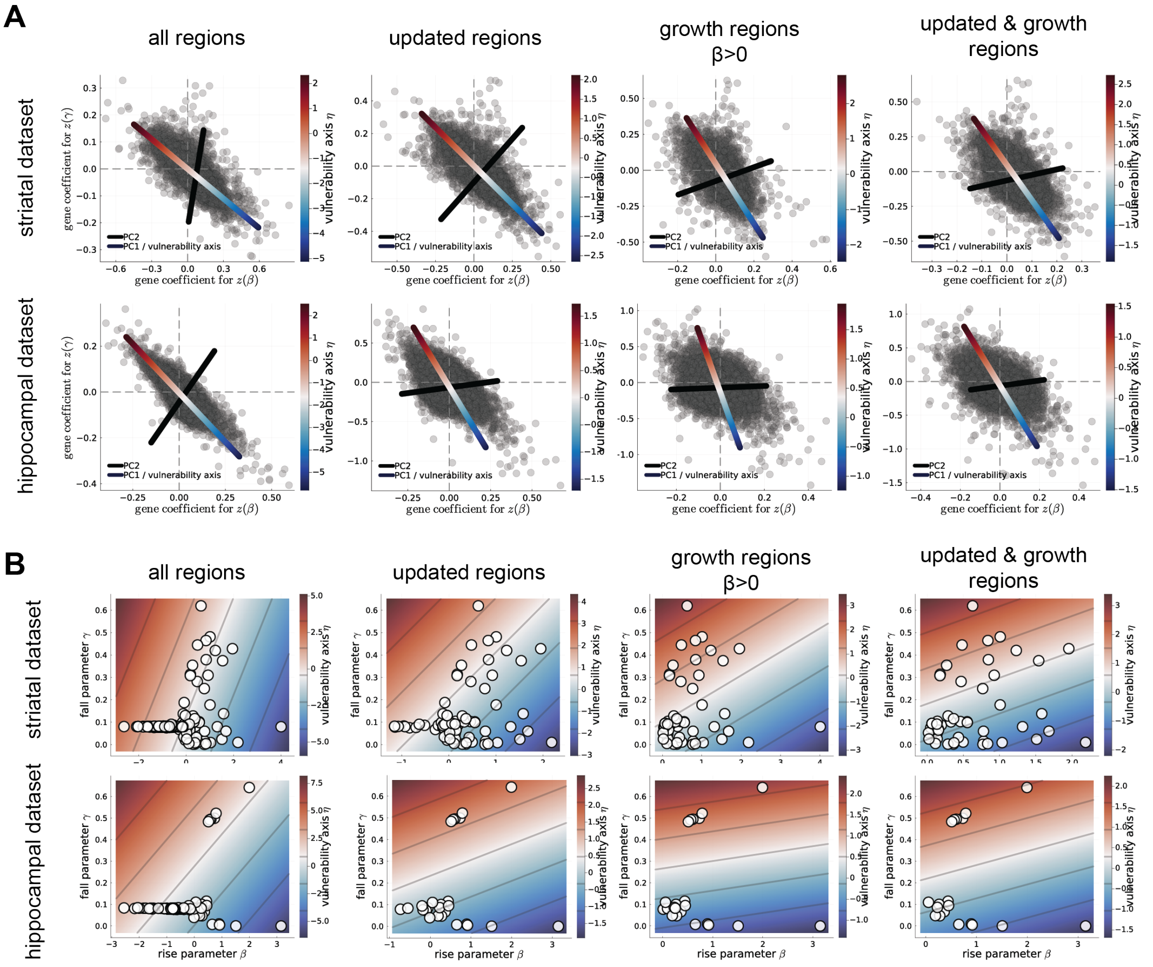

Figure 2: Effect of filtering regions on vulnerability axis. (**A**) PCA components of the gene-parameter correlations for varying filtering stages of the brain regions. Updated regions exclude region's whose parameter posteriors are not updated from their priors. (**B**) Corresponding PC1/vulnerability axis of mapped on to the raw model parameter space with brain regions (white dots).

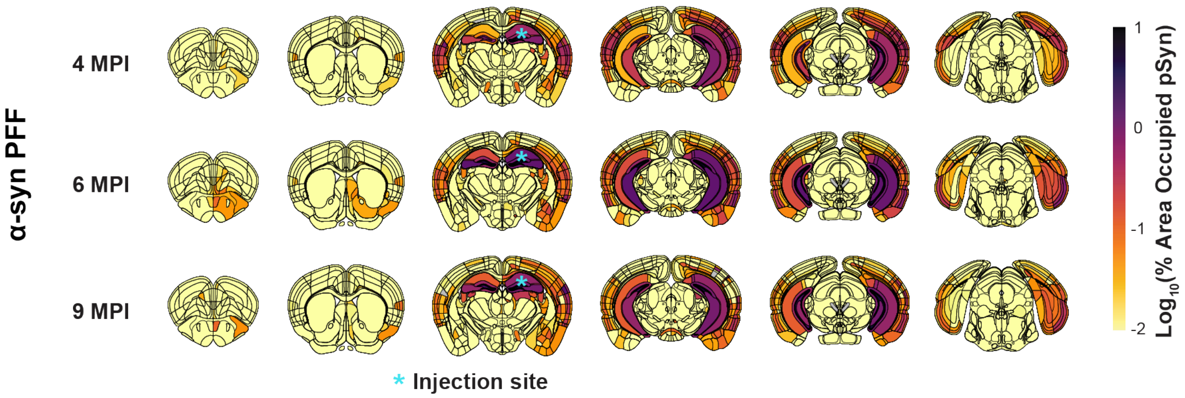

Figure 3: α-Synuclein pathology distribution following hippocampal injection of α-synuclein PFFs. Wildtype mice injected with α-synuclein PFFs in the hippocampus and overlaying cortex were aged 4, 6, or 9 MPI. Anatomical heatmaps showing the log-transformed percentage of area occupied by phosphorylated α-synuclein. The injection site is indicated by an asterisk.

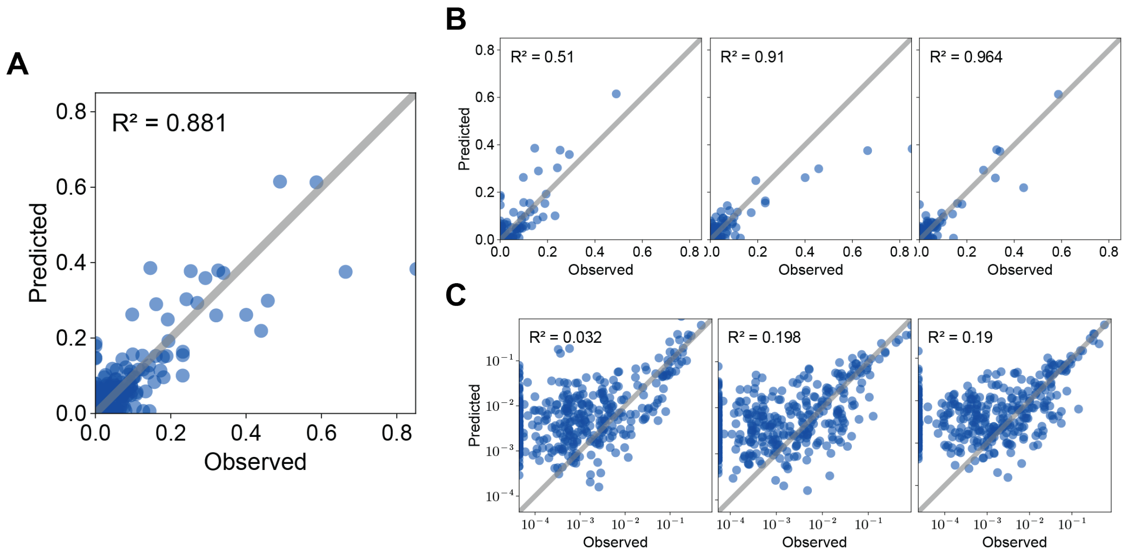

Figure 4: Model performance of DIFF-RF on hippocampal dataset. (**A**) Predicted versus observed pathology for each model, pooling all regions and timepoints. Each point corresponds to a region–timepoint pair; the diagonal indicates identity. (**B**) Predicted versus observed pathology at individual timepoints (all regions pooled). Panels correspond to increasing time after injection (0.1 to 9 months). The diagonal indicates identity. (**C**) Same as panel (B) but on log-log scales.

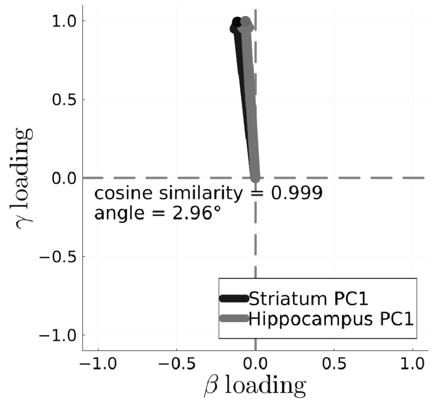

Figure 5: Vulnerability axes comparison across datasets. Comparison of the PC1 / vulnerability axes found in the striatal and hippocampal dataset in raw (β_i, γ_i) space.

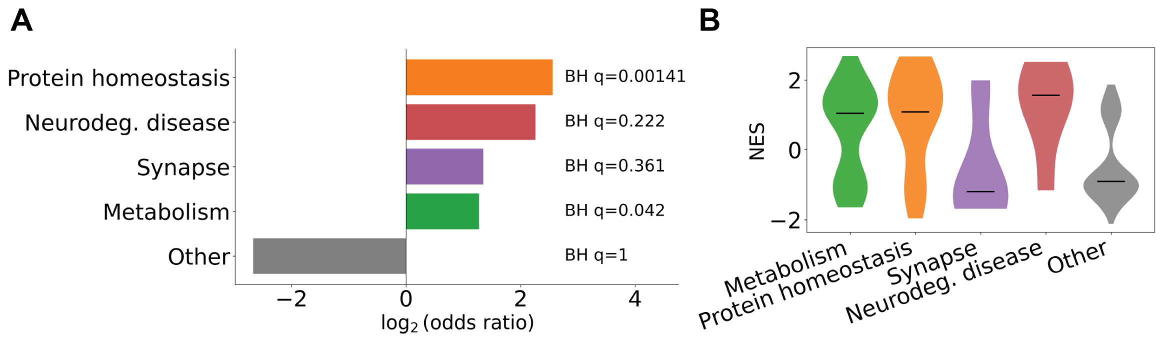

Figure 6: Additional analyses of gene enrichment for the hippocampal dataset. (**A**) Enrichment of functional gene categories along the vulnerability axis, shown as $\log_2$ odds ratios with Benjamini–Hochberg adjusted q-values. (**B**) Distribution of normalized enrichment scores (NES) across functional categories.

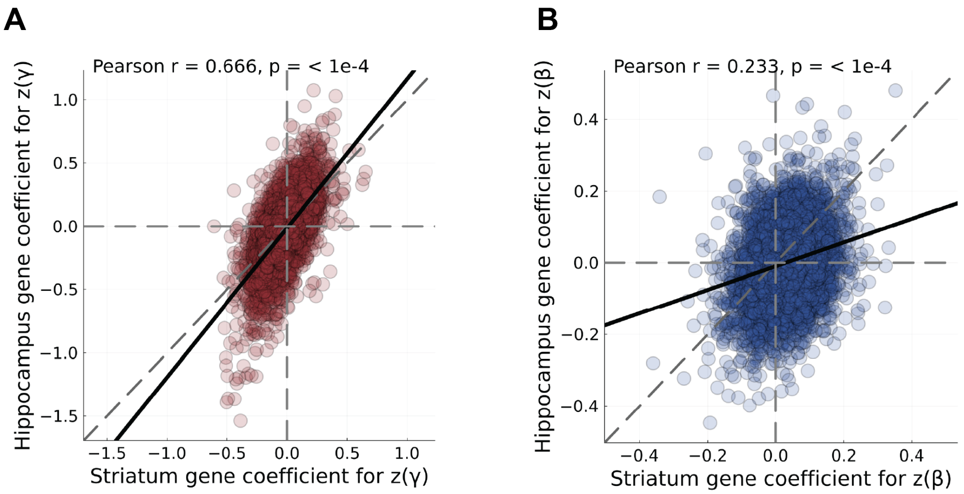

Figure 7: Reproducibility of gene-parameter associations. (**A**) Comparison of gene-parameter correlation coefficients in the striatal and hippocampal dataset for the fall parameter γ_i. (**B**) Same as in panel (A) but for the fall parameter β_i.

Table 1: Keyword-based mapping used to assign KEGG pathways to broad functional categories for summary analyses. Pathways were matched case-insensitively to the first category whose keyword list was satisfied; pathways that matched none of the listed keywords were assigned to *Other*.

**Category**	**Keywords used for assignment**
Metabolism	`metabolism, metabolic, oxidative phosphorylation, mitochond, glycolysis, tca, citrate cycle, respiratory chain, fatty acid, lipid, cholesterol, biosynthesis, amino acid, nucleotide, energy`
Protein homeostasis	`proteasome, ubiquitin, ubiquitination, autophagy, lysosome, endoplasmic reticulum, er , chaperone, protein folding, protein processing, ribosome, translation, mrna, degradation, unfolded protein, quality control, mitophagy`
Synaptic function	`synapse, synaptic, dopaminergic synapse, glutamatergic synapse, gabaergic synapse, neurotransmitter, vesicle, long-term potentiation, long-term depression`
Neurodegenerative disease	`parkinson, alzheimer, huntington, prion, amyotrophic, neurodegenerative, als`
Other	Any pathway name not matching the keyword lists above

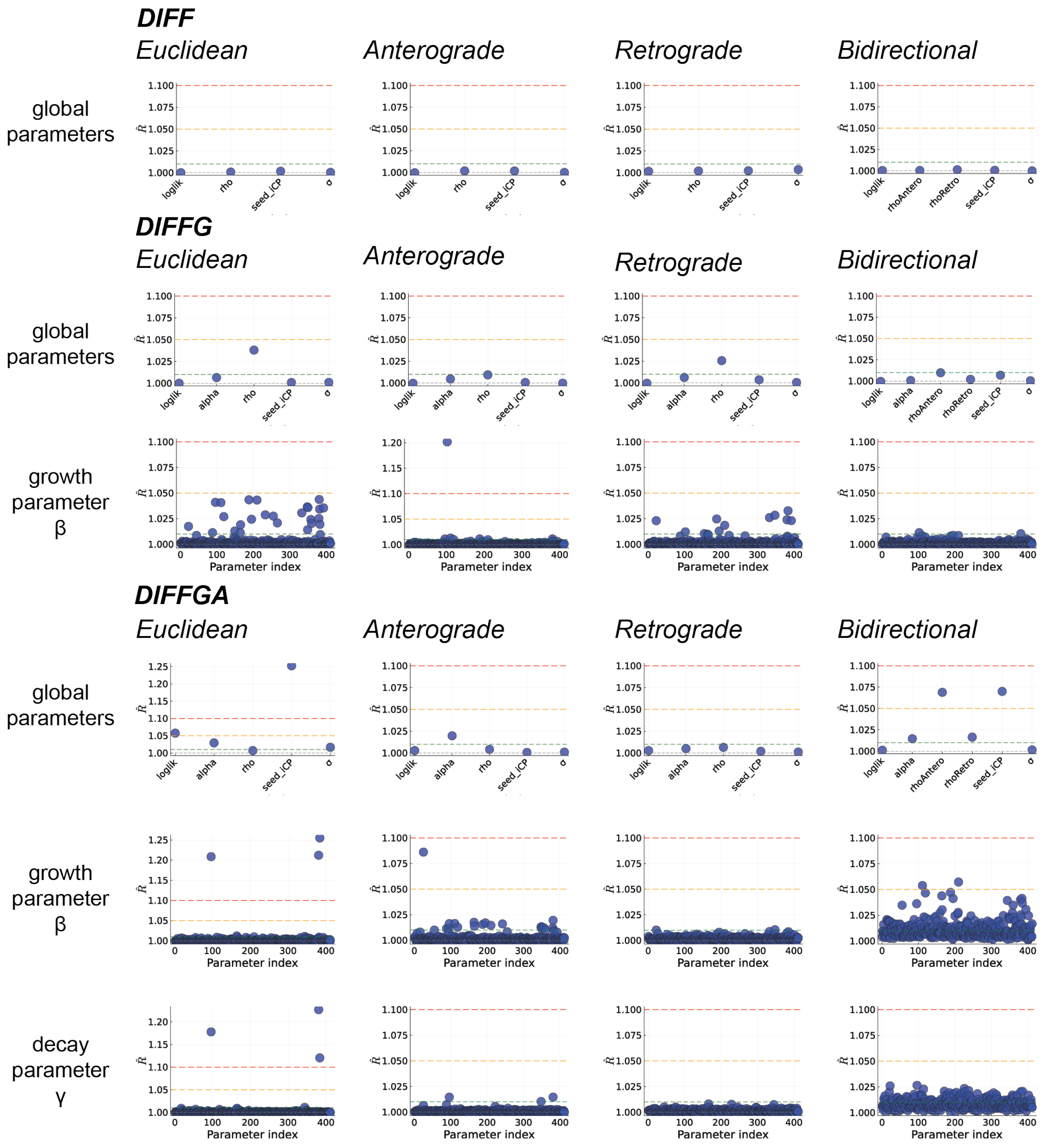

Figure 8: Convergence diagnostics across models and datasets. Gelman–Rubin statistics ($\hat{R}$) for all inferred parameters across model variants and datasets. Each panel shows $\hat{R}$ values either aggregated by parameter type (top rows) or for all individual parameters indexed sequentially (rows labeled "Parameter index"). Horizontal dashed lines indicate common convergence thresholds ($\hat{R} = 1.01$, 1.05, and 1.10).

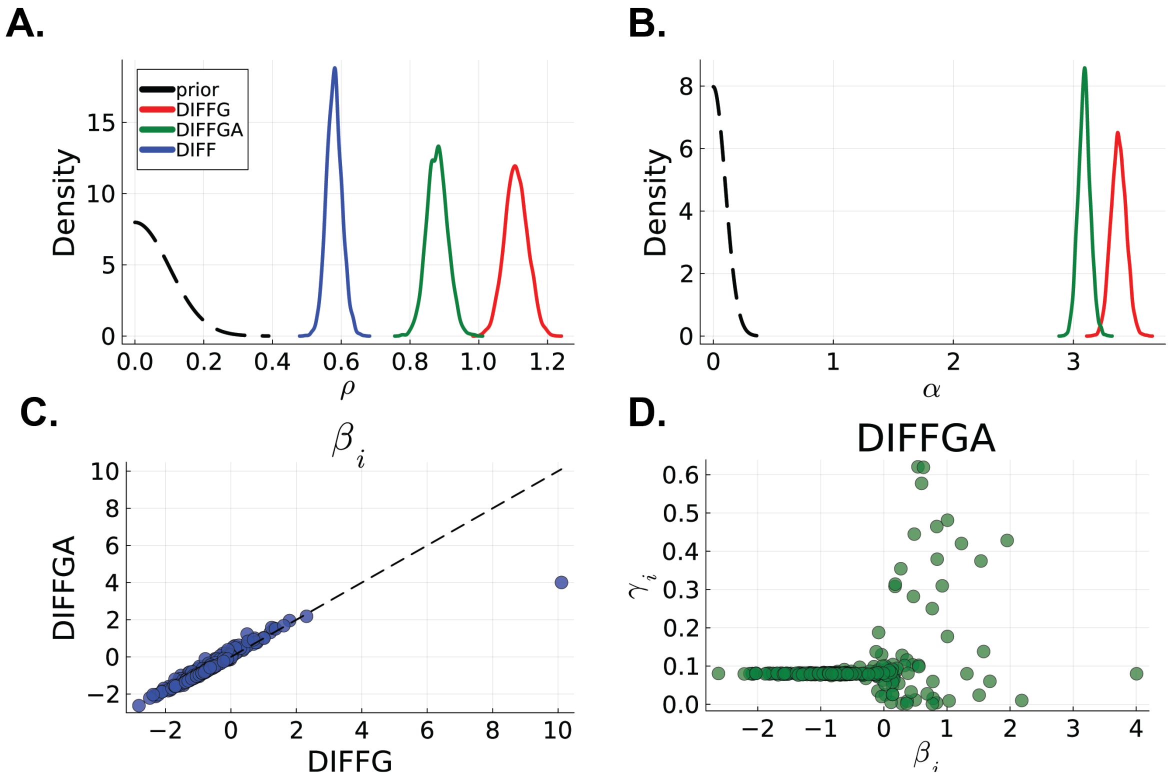

Figure 9: Prior and posterior distributions and regional parameter relationships. (A) Prior (black dashed) and posterior densities for the global transport parameter ρ across model classes (DIFF, DIFFG, DIFFGA). (B) Prior (black dashed) and posterior densities for the global growth parameter α in DIFFG and DIFFGA. (C) Comparison of the posterior means of the regional carrying capacity parameters β_i inferred under DIFFG and DIFFGA; the dashed line denotes identity. (D) Means of the posterior regional decay parameters γ_i versus carrying capacity parameters β_i in DIFFGA.
